# Genome-wide association study of untargeted plasma metabolomic profiles identifies host genetic regulation in people with HIV

**DOI:** 10.1016/j.xhgg.2026.100635

**Published:** 2026-06-17

**Authors:** Mariam Ait Oumelloul, Adriaan van der Graaf, Simon Tang, Christian W. Thorball, Marco Labarile, Ali Saadat, Valeriia Timonina, Isabella C. Schöpf, Gilles Wandeler, Johannes Nemeth, Matthias Cavassini, Alexandra Calmy, Patrick Schmid, Marcel Stöckle, Luigia Elzi, Nicola Zamboni, Roger D. Kouyos, Philip E. Tarr, Jacques Fellay, Swiss HIV Cohort Study

**Affiliations:** 1 School of Life Sciences, Ecole Polytechnique Fédérale de Lausanne, Lausanne, Switzerland; 2 Swiss Institute of Bioinformatics, Lausanne, Switzerland; 3 Department of Computational Biology, University of Lausanne, Lausanne, Switzerland; 4 Precision Medicine Unit, Biomedical Data Science Center, Lausanne University Hospital and University of Lausanne, Lausanne, Switzerland; 5 Division of Infectious Diseases and Hospital Epidemiology, University Hospital Zurich, Zurich, Switzerland; 6 Institute of Medical Virology, University of Zurich, Zurich, Switzerland; 7 University Department of Medicine and Infectious Diseases Service, Kantonsspital Baselland, University of Basel, Bruderholz, Switzerland; 8 Department of Visceral Surgery and Medicine, Inselspital, Bern University Hospital, University of Bern, Bern, Switzerland; 9 Institute of Social and Preventive Medicine, University of Bern, Bern, Switzerland; 10 Department of Infectious Diseases, Inselspital, Bern University Hospital, University of Bern, Bern, Switzerland; 11 Infectious Diseases Service, Lausanne University Hospital, University of Lausanne, Lausanne, Switzerland; 12 HIV/AIDS Unit, Division of Infectious Diseases, University Hospital Geneva, Geneva, Switzerland; 13 Faculty of Medicine, University of Geneva, Geneva, Switzerland; 14 Division of Infectious Diseases, Cantonal Hospital St Gallen, St Gallen, Switzerland; 15 Division of Infectious Diseases and Hospital Epidemiology, University Hospital Basel, University of Basel, Basel, Switzerland; 16 Malattie Infettive, Ospedale Regionale di Bellinzona e Valli, Bellinzona, Switzerland; 17 Institute of Molecular Systems Biology, ETH Zürich, Zürich, Switzerland; 18 Swiss Multi-Omics Center, Zurich, Switzerland

**Keywords:** people with HIV, genomics, GWAS, metabolomics, circulating metabolites, multi-omics, aging-related comorbidities

## Abstract

People with human immunodeficiency virus (PWH) exhibit accelerated aging and a higher prevalence of aging-related conditions, despite effective antiretroviral therapy. The biological mechanisms involved remain incompletely understood. Integrating genomic and metabolomic profiling may help uncover genes and pathways contributing to aging-related disease in this population.

Using a genome-wide association study framework and untargeted metabolomic profiling, we searched for associations between human genetic variants and the plasma concentrations of 1,930 putative metabolites in 1,244 individuals enrolled in the Swiss HIV Cohort Study. We performed an expression quantitative trait locus (eQTL) colocalization analysis to explore biological links between genetic variants and metabolites and used Mendelian randomization to search for causal relationships between metabolites and aging-related diseases.

We identified 27 metabolites significantly associated with 12 genetic loci, including genes encoding the metabolic enzymes *NAT8* and *FUT2*; 10 associations had been previously reported in general-population studies, of which eight were replicated in our analysis. The colocalization analyses provided evidence for a large overlap between genetic regulation of mRNA expression and metabolite levels, while Mendelian randomization suggested several causal effects.

Our study uncovered genetic-metabolic associations observed in PWH and explored their biological relevance. These findings highlight the potential of integrated multi-omics profiling to deepen mechanistic understanding and inform future precision approaches to comorbidity management in this population.

## Teaser

We investigated how human genetic variation influences circulating metabolites in people with HIV. By integrating genetics and metabolomic data from the Swiss HIV Cohort Study, we identified genetic regulators of multiple metabolites and explored their potential links to aging-related disease.

## Introduction

Human immunodeficiency virus (HIV) remains a major global health challenge, with over 42 million individuals currently living with the virus.^1^ The administration of antiretroviral therapy (ART) in people with HIV (PWH) demonstrates notable efficacy in suppressing the virus and restoring immune function. Consequently, the life expectancy of PWH receiving optimal treatment has substantially increased, now approaching that of the general population. Despite these advances, PWH exhibit a disproportionately high prevalence of aging-related comorbidities, such as cardiovascular,^2^^,^^3^^,^^4^ kidney,^5^ and liver diseases,^6^^,^^7^ compared to general populations. These elevated risks suggest complex underlying biological mechanisms that extend beyond viral suppression, underscoring the need for comprehensive molecular investigation.

Metabolomics is a powerful analytical approach to characterize the molecular landscape of complex physiological processes. Through systematic profiling of low-molecular-weight molecules in biological specimens, this approach provides unprecedented insights into the intricate interplay between genetics, environmental factors, and molecular perturbations underlying health and disease.^8^^,^^9^ Metabolites are considered proximal reporters of disease due to their abundance in biological specimens, often being directly related to disturbed physiological functions and pathogenic mechanisms.^10^ In the context of HIV, metabolomics studies suggest that ART only partially restores the metabolic disturbances caused by HIV. Moreover, ART itself has been associated with the onset of additional metabolic regulations.^11^^,^^12^ A recent work in the Swiss HIV Cohort Study (SHCS) highlighted distinct metabolomic signatures linked to ART exposure in PWH.^13^

The integration of metabolomics and genetics offers a promising approach to characterizing metabolic alterations that may contribute to the development of aging-related comorbidities in PWH. Indeed, genetic factors are known to play a critical role in shaping the metabolome, with several genome-wide association studies (GWASs) published providing key insights into the genetic regulation of systemic metabolism in both healthy and disease cohorts. For instance, studies in healthy populations have demonstrated how genetic variants influence metabolite levels. Notably, early work by Gieger et al. established that the functional characterization of genes associated with specific metabolites could reveal underlying biological processes.^14^ More recent studies, such as those from the Canadian Longitudinal Study on Aging (CLSA)^15^ and the 500FG cohort,^16^ have expanded this understanding by identifying additional gene-metabolite associations and exploring their potential implications for disease risk and treatment strategies. In disease-specific contexts, genetics has been employed to identify metabolites that may play causal roles in conditions such as multiple sclerosis,^17^ irritable bowel syndrome,^18^ and chronic kidney disease,^19^^,^^20^ offering new avenues for biomarker discovery and therapeutic intervention.

**Figure 1:**
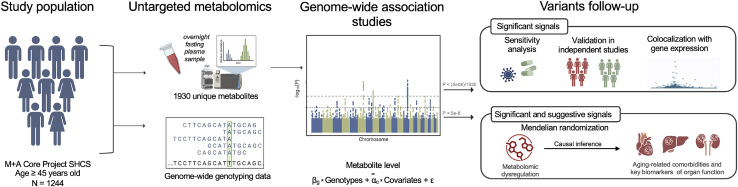
Overview of the study design Schematic representation of the genome-wide screens for plasma metabolite levels and their follow-up analyses. The study population included 1,244 participants from the SHCS Metabolism + Aging (M + A) Core Project (age ≥45 years). Overnight fasting plasma samples were profiled by untargeted metabolomics. Genome-wide association studies (GWASs) were performed for each metabolite detected. Variants reaching a metabolome-wide significance threshold (*p* < 5 × 10^−8^/1,930) were prioritized for follow-up, including sensitivity analyses, validation in independent studies, and colocalization with gene-expression signals. In addition, significant and suggestive GWAS signals (*p* < 5 × 10^−8^) were carried forward to MR analyses to evaluate potential causal effects of metabolite variation on aging-related comorbidities and key biomarkers of organ function. Figure partially created with BioRender.com.

This study aims to investigate the genetic influences on the plasma metabolome of PWH with a focus on understanding their potential contribution to aging-related conditions. We performed a large-scale GWAS of plasma levels of 1,930 putative metabolites, measured using an untargeted metabolomics approach in 1,244 individuals from the SHCS to characterize the genetic architecture of metabolic traits in this population. Building on the identification of specific genetic variants that impact metabolite levels, we then used Mendelian randomization to infer the causal effects of these putative metabolites on comorbidities such as cardiovascular, kidney, and liver diseases and their key biomarkers (Figure 1).

Our hypothesis-free approach provides biological insights into metabolic pathways potentially linked to aging-related comorbidities in PWH, offering a basis for future studies aimed at improving clinical care and health outcomes in this population.

## Material and methods

Ethical approval declarations are as follows. The SHCS was approved by the ethics committees of the participating institutions: BASEC-Nr. 2023-02080, Kantonale Ethikkommission Zürich; Ethikkommission Nordwest- und Zentralschweiz EKNZ; Kantonale Ethikkommission Bern; Commission Cantonale d’éthique de la recherche sur l’être humain CCER-GE; Commission cantonale d’éthique de la recherche sur l’être humain, CER-VD; Comitato etico cantonale, Ticino; Ethikkommission Ostschweiz EKOS. Written informed consent was obtained from all participants.

### Study participants

The SHCS is a nationwide, prospective multicenter cohort study with semiannual visits and blood collections, which has enrolled over 20,000 HIV-infected adults living in Switzerland since the late 1980s.^21^ Among these, 7,460 individuals have undergone genome-wide genotyping. From this genotyped group, we included 1,244 participants in the Metabolism and Aging (M + A) Core Project, a sub-study focused on assessing circulating plasma metabolites.^13^ Inclusion criteria for the M + A Project were age ≥45 years and the ability to undergo neurocognitive testing in German, French, Italian, or English and to provide a plasma sample after an overnight fast. Information on aging-related comorbidities, including diabetes mellitus type II, chronic kidney disease, liver fibrosis, and major cardiac events, was collected at the time of metabolomic profiling, along with key circulating biomarkers relevant to metabolic, hepatic, renal, and cardiovascular function. Detailed definitions for each are provided in the supplemental methods.

### Genotyping and ancestry inference

Genome-wide genotyping data were obtained using various genotyping arrays from Illumina based on of previous SHCS studies. Each genotyping batch was imputed separately using the 1000 Genomes Project (1kGP),^22^^,^^23^ then filtered to keep genetic variants with an imputation quality score INFO > 0.8 prior to merging. The chromosome positions were aligned to the Human Build 37 (GRCh37).

We inferred genetic ancestry by combining the genotypes of the SHCS population with genotypes of the 1kGP. Using PLINK,^24^ principal-component analysis (PCA) on this combined genotype panel was then used to detect population structure (Figure S1).

### Metabolite profiling and data processing

Fasting plasma metabolites had been previously quantified in the context of the M + A SHCS metabolomics project^13^ using an untargeted flow-injection time-of-flight mass spectrometry platform,^25^ yielding 2,310 molecular formulas corresponding to up to 1,930 putative metabolites,^26^ including xenobiotics, drugs, and their relative metabolites (detailed extraction protocols, instrument parameters, and formula-to-metabolite and mapping procedures are provided in the supplemental methods).

We used the human metabolome database version 5.0 (HMDB)^27^ to categorize metabolites into specific superpathways including lipids/lipid-like molecules, amino acids/peptides, carbohydrates, and nucleosides/nucleotides. Metabolites not assigned to one of HMDB superpathways were evaluated for potential xenobiotic classification using curated drug- and exposure-related databases accessed through the MBROLE3 server.^28^ Details regarding the complete list of databases and annotation criteria used for xenobiotic classification are provided in the supplemental methods. Compounds without matches were assigned to the “other” category.

### Statistical analysis

#### Associations between metabolites and other variables

To characterize the intrinsic structure of the metabolome, we first computed pairwise Spearman correlations between metabolites using values adjusted for age, sex assigned at birth, and smoking status (these corrected intensities were used solely for unsupervised clustering analyses).

In a separate analysis, we evaluated demographic influences on individual metabolites using univariate logistic regression models, testing associations with sex assigned at birth, smoking status, reported ethnicity, and genetically inferred ancestry. *p* values were adjusted for multiple testing using a Bonferroni threshold and considered significant at *p* < 0.05.

#### Heritability of metabolite levels

The heritability of metabolite plasma levels was evaluated using the GCTA-GREML software tool.^29^ Specifically, all analyzed variants on autosomes with a minor allele frequency (MAF) ≥0.05 were incorporated into the genetic relatedness matrix (GRM) calculation. This GRM was subsequently utilized to estimate the variance using default settings.

We used the GCTA-GREML statistical power approach,^30^ with default parameters, to assess the statistical power to detect non-zero SNP-based heritability (*h*^2^ > 0) at the specified type I error rate. Based on this framework, our study had sufficient statistical power to detect significant non-zero SNP-based heritability for the 1,409 putative metabolites. We also evaluated the precision of the heritability estimates using the heritability *Z* score, defined as *h*^2^/SE.

#### GWASs of metabolite levels

For the GWAS, metabolite levels were log transformed to achieve a more symmetric distribution. Subsequently, outliers were identified and removed using Tukey’s fences method, where metabolite intensities falling below *Q*_1_ − 1.5 × IQR (where IQR is interquartile range) or above *Q*_3_ + 1.5 × IQR were considered outliers. Finally, the data were standardized to have a mean of 0 and a standard deviation of 1.

GWASs were performed using REGENIE (v3.2.5.3) in a two-step framework that incorporates whole-genome prediction and leave-one-chromosome-out modeling to account for population structure.^31^ High-quality genotyped variants were used in step one, and association testing in step two was conducted on imputed variants following standard filtering and quality-control procedures. Following data processing and quality control, GWAS analyses using linear regression for metabolites were conducted, accounting for covariates including age at the time of metabolomic profiling, sex assigned at birth, smoking status, genotyping batches, and the first 10 genetic principal components. Detailed GWAS parameters, variant-filtering criteria, and model specifications are provided in the supplemental methods.

The genomic inflation factor for each GWAS result was calculated as the median of the observed chi-squared test statistics divided by the median of the expected chi-squared test statistics for each putative metabolite.

We used Bonferroni correction for multiple testing; associations with a *p* value smaller than 2.59 × 10^−11^, corresponding to 5 × 10^−8^/1,930, were considered genome-wide significant.

To identify conditionally independent SNPs from the GWAS, conditional and joint (COJO) analysis in GCTA^32^^,^^33^ was used, which leverages linkage disequilibrium (LD) estimates between SNPs and summary statistics. For each putative metabolite, the SNP with the lowest association *p* value was designated as the index SNP, and a metabolite quantitative trait locus was defined following the approach used by FUMA for genomic risk loci.^34^

#### Sensitivity analysis

To evaluate the impact of sample diversity on the association results, we performed additional sensitivity analyses. These included reanalysis of participants clustered with European ancestry from the 1kGP to assess consistency within a more genetically homogeneous population, as well as a sex-stratified GWAS restricted to males to assess the stability of genetic effect estimates in the largest demographic subgroup. In a separate model, we incorporated HIV-specific contextual variables as covariates, including the average CD4-positive T cell count and HIV-1 viral load calculated over the 6 months preceding metabolomic profiling, the ART regimen (categorized as protease inhibitor-based, non-nucleoside reverse transcriptase inhibitor-based, integrase strand transfer inhibitor-based, or other), duration of HIV infection, and the duration on the combination therapy at the time of metabolomic profiling. The duration of HIV infection is defined based on the documented date of the first positive test, or the date of the registration visit of the participant to the cohort if the former is not available. To explore potential synergic effects, we conducted exploratory interaction analyses testing SNP × HIV-specific contextual variables. Interaction terms were added to linear regression models applying the same covariate structure as used in the primary REGENIE analyses to ensure methodological consistency.

#### Comparison with previously reported metabolite-QTL associations

To contextualize GWAS findings and determine whether the identified metabolite-variant associations had been previously reported, we systematically compared each significant locus with previously reported metabolite quantitative trait loci (QTLs) from large mass-spectrometry-based studies (Shin et al.,^35^ Long et al.,^36^ Lotta et al.,^37^ Hysi et al.,^38^ Yin et al.,^39^ Schlosser et al.,^20^ and Chen et al.^15^). The cohort size, the number of metabolites tested, and the genetic variants reported in these studies are summarized in the additional files. Harmonization procedures, matching criteria, and comparison workflows are detailed in the supplemental methods.

#### eQTL colocalization analysis

We performed expression QTL (eQTL) colocalization analysis to investigate whether the genome-wide significant genetic variants were associated with changes in gene expression across different tissues using data from the 2023 eQTL Catalogue.^40^^,^^41^^,^^42^ For each gene, SNPs within a ±500-kbp window of an mGWAS hit were tested for colocalization against eQTLs using the coloc v5.2.3 package.^43^ The metabolites that had PP.H4 ≥ 0.8(posterior probabilities of two traits share one causal SNP) with eQTL were considered to pass the colocalization test. Detailed parameters and window definitions are provided in the supplemental methods.

#### Mendelian randomization

##### Study design and outcome selection

Mendelian randomization (MR) leverages genetic variants associated with an exposure to infer its causal effect on outcomes.^44^ We applied two-sample MR to investigate potential causal effects of circulating plasma metabolites on biomarkers and diagnoses relevant to major aging-related comorbidities, with a particular focus on cardiovascular, kidney, liver, and neurodegenerative conditions. To capture complementary dimensions of disease biology, we included both continuous biomarkers, which may reflect subclinical or intermediate physiological changes, and binary disease outcomes, which represent clinically manifest conditions. We used large GWAS datasets from the UK Biobank (UKB) database^45^ and additional publicly available studies, considering a total of 18 outcomes. We included, in total, 11 biomarkers associated with kidney function (creatinine), liver function (albumin, bilirubin, *γ*-glutamyl transferase, alanine aminotransferase, and aspartate aminotransferase), cardiovascular function (total cholesterol, triglycerides, and high-density lipoprotein [HDL]), bone metabolism (estimated bone mineral density [eBMD]),^46^ and biological aging (telomere length).^47^ Additionally, we included seven diagnoses based on their relevance to aging-related comorbidities: diabetes mellitus type II,^48^ chronic kidney disease,^49^ stroke,^50^ coronary artery disease,^51^ Alzheimer disease,^52^ Parkinson disease,^53^ and liver cirrhosis.^54^ Details on the specific traits and locations of the GWAS data for these outcomes are provided in Table S12.

##### Exposure selection

Regarding the exposures, we included all metabolites that showed genome-wide significant associations with at least one genetic locus (*p* < 5 × 10^−8^), resulting in a total of 187 metabolites. We performed LD clumping to retain independent genetic instruments, excluding SNPs with *r*^2^ ≥ 0.001 within a 1-Mb window and retaining the variant with the smaller *p* value. For metabolites instrumented by a single SNP, we used the SNP-specific approximate *F* statistic, calculated as *F* = *β*^2^/SE^2^, where *β* is the SNP effect on the metabolite and SE is its standard error. For metabolites instrumented by multiple independent SNPs, we used the overall instrument-set *F* statistic, calculated as *F* = [(*n* − *K* − 1)/*K*] × [*R*^2^/(1 − *R*^2^)], where *n* is the exposure GWAS sample size, *K* is the number of SNPs in the instrument, and *R*^2^ is the total variance in the metabolite explained by the retained SNPs.^55^ The variance explained by each SNP was estimated as $R_{j}^{2}=2f\left(1-f\right)\beta^{2}$, where *f* is the effect allele frequency. Total *R*^2^ was obtained by summing $R_{j}^{2}$ across independent SNPs. To reduce the risk of weak-instrument bias, instruments with *F* > 10 were considered sufficiently strong.

##### MR analysis

We conducted the analyses using MR-link-2 (https://github.com/adriaan-vd-graaf/mrlink2).^56^ MR-link-2 is a summary statistics MR method that estimates causality and pleiotropy from single associated regions, requiring only summary statistics of an exposure and an outcome, along with a genotype reference file. The genotype reference was generated from SHCS cohort filtering for individuals of European genetic ancestry. A significance threshold of 1.48 × 10^−5^ was applied, corresponding to a Bonferroni correction for multiple testing (0.05 divided by the total number of metabolite-trait relationships assessed: *n* = 3366). In addition to estimating the causal effect, MR-link-2 reports a parameter (sigma) that captures residual horizontal pleiotropy within the tested region. This parameter was examined to evaluate the potential contribution of pleiotropic effects to the observed associations.

We additionally performed bidirectional MR and applied Steiger filtering to test for the correct instrument orientation. Finally, to identify causal relationships unique to the PWH cohort compared to the general population, we examined the causal effects of 214 overlapping plasma metabolites in the SHCS from the CLSA^15^ cohort on the same outcomes in the UKB. An overview of the analytical workflow for the two-sample MR analyses is shown in Figure S2.

## Results

### Characterization of the plasma metabolome

We quantified 1,930 putative metabolites in 1,244 individuals by untargeted mass spectrometry from blood fasting plasma samples from the M + A Core Project of the SHCS. The study population was predominantly male (79.90%) and of European ancestry (90.03%), with a median age of 55 years. Most participants were receiving ART, with protease inhibitor-, non-nucleoside reverse transcriptase inhibitor (NNRTI)-, and integrase strand transfer inhibitor (INSTI)-based regimens all represented (20.21%, 31.93%, and 26.11%, respectively). The median CD4 cell count at sampling was 643 cells/μL, and only 5.75% of individuals had an HIV-1 RNA level exceeding 50 copies/mL, indicating that the vast majority were virologically suppressed at the time of metabolomic profiling. Participants had been diagnosed with chronic kidney disease, liver fibrosis, cardiovascular events, and type II diabetes mellitus prior to metabolomic profiling, each affecting approximately 9%–15% of the cohort (Table 1). Detailed cohort characteristics of the SHCS are described in Scherrer et al.,^21^ whereas metabolite information is shown in Table S1).

**Table 1:** Characteristics of the study populations

Characteristic	SHCS (M + A) core project (*N* = 1,244)
Age (years)	55 (51; 61)
Sex at birth (male)	993 (79.90%)
Ethnicity∗ (European)	1,120 (90.03%)
Current smoking (yes)	437 (35.13%)
Time since HIV infection (years)	17.44 (10.32, 34.52)
Viral RNA load >50 (copies/mL)	70 (5.75%)
CD4 [cells/μL]	643 (479.5; 840.0)
ART	–
PI	250 (20.21%)
NNRTI	395 (31.93%)
INSTI	323 (26.11%)
Other	256 (21.75%)
Missing	13 (1.04%)
Chronic kidney disease	109 (8.76%)
Liver fibrosis	145 (11.66%)
Cardiovascular events	123 (9.89%)
Diabetes mellitus type II	185 (14.87%)

Data are shown as a median (IQR) for continuous variables and *n* (%) for categorical variables. Clinical diagnoses are documented prior to the date of metabolomic sampling. ART, antiretroviral therapy; PI, protease inhibitor; NNRTI, non-nucleoside reverse transcriptase inhibitor; INSTI, integrase strand transfer inhibitor; CD4, CD4-positive T-lymphocyte count, IQR, interquartile range.

**Figure 2:**
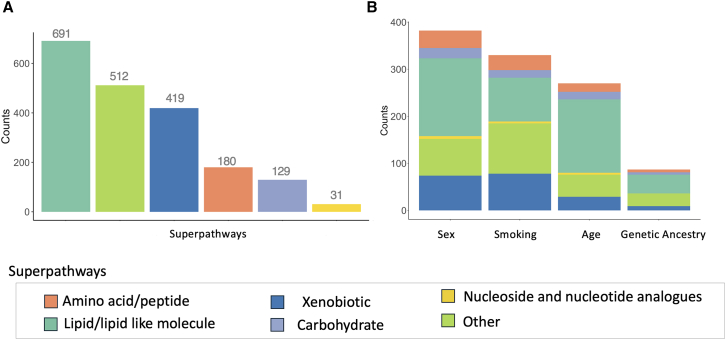
Distribution of metabolites across superpathways and covariate associations (A) Bar plot showing the number of metabolites assigned to each superpathway. (B) Stacked bar plots showing the number of metabolites significantly associated with covariates (sex assigned at birth, smoking, age, genetic ancestry), stratified by superpathway category. Color coding represents metabolite superpathways.

Of all putative metabolites detected in our samples, we classified 1,418 across five superpathways (lipids and lipid-like molecules, xenobiotics, amino acids/peptides, carbohydrates, and nucleosides/nucleotides) using public databases.^27^^,^^28^ We grouped 512 (26.53%) metabolites not matching these predefined categories as “other.” The majority of the detected metabolites were lipids (*n* = 691, 35.80%), followed by xenobiotics (*n* = 419, 21.71%), which were defined as chemical compounds that are foreign to living organisms, highlighting the significant presence of both endogenous and exogenous compounds in this cohort (Figure 2A).

To evaluate metabolic relationships within and across superpathways among metabolites, we calculated Spearman’s correlation coefficients between metabolites (Figure S3). A moderate within-lipid superpathway correlation was observed, particularly among glycerophospholipids, which frequently clustered with saccharolipids, suggesting closely related metabolic functions. In contrast, inter-superpathway correlations were more pronounced among the other metabolite superpathways. We used linear regression models to evaluate univariate demographic associations (Figure 2B; Table S2). Of the 1,930 detected putative metabolites, 19.79% (*n* = 382) were significantly associated with sex assigned at birth, 17.10% (*n* = 330) with smoking status, 14% (*n* = 270) with age, and 4.51% with genetic ancestry (*n* = 87). Smoking showed the highest proportion of xenobiotic associations (Figure 2B); notably, we detected a feature with the formula *C*_8_*H*_8_*O*_4_*S*, which matched the HMDB entry for 4-vinylphenyl sulfate—a metabolite previously associated with smoking.^57^

We computed the heritability of each putative metabolite plasma level to estimate the total variance explained by genetic variation through a mixed model approach.^29^ 582 metabolites had a significant heritability (FDR < 0.05). The median SNP-based heritability of these putative metabolites was 19.76%. Heritability was the highest for lipids (median = 21.01%), indicating a strong genetic influence on their concentrations. The top three putative metabolites with the highest heritability were an N-Ac-hexosamine (${\hat{h}}^{2}$: 76%, ${\hat{h}}^{2}/\text{SE}$ = 3.65), N-acetylcitrulline (${\hat{h}}^{2}$: 74%, ${\hat{h}}^{2}/\text{SE}$ = 4.02), and cytidine diphosphate-ethanolamine (${\hat{h}}^{2}$: 69%, ${\hat{h}}^{2}/\text{SE}$ = 3.56) (Figure S4 and Table S3). Some metabolites nevertheless exhibited lower heritability *Z* scores, indicating reduced precision of the corresponding SNP-based heritability estimates and therefore lower confidence in the exact magnitude of their estimated genetic contribution, likely due to the relatively small sample size of our analysis.

### Genome-wide associations of blood metabolites

**Figure 3:**
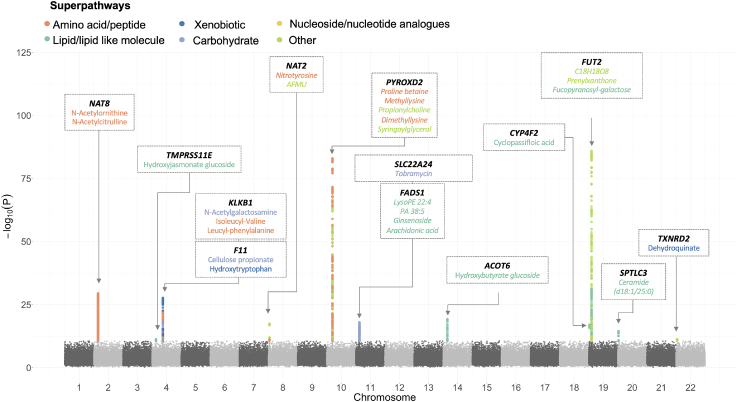
Manhattan plot of genome-wide associations of blood metabolites Manhattan plot displaying chromosomal positions (*x* axis) of significant associations (*p* < 2.59 × 10^−11^, accounting for multiple testing, *y* axis). Colored points represent metabolome-wide significant associations, with colors indicating metabolite superpathways; gray points are shown only as background. *p* values were obtained from genome-wide summary statistics from linear regression models using genetic variants as predictors and metabolite levels as outcomes. Closest genes identified for corresponding loci are annotated.

We performed GWAS of the plasma concentrations of the 1,930 putative metabolites detected in our study population. We identified 12 genome-wide significant loci associated with 27 putative metabolites after applying Bonferroni correction for the number of independent SNPs and the total number of metabolites tested (*p* < 5 × 10^–8^/1930 = 2.59 × 10^−10^) (see section material and methods) (Figure 3 and Table S4). Assessment of genomic inflation factors revealed no evidence of excessive test statistic inflation or population stratification (median lambda = 1.00; Table S5).

GWAS across all the putative metabolite species revealed the strongest associations in the *FUT2* and *PYROXD2* gene regions. We also identified several pleiotropic regions (i.e., regions that affected multiple metabolite measurements), notably a locus on chromosome 11 harboring the fatty acid desaturase (*FADS*) gene family, which was associated with five metabolites, including four lipids.

To ensure our results were not biased by the multi-ancestry nature of our cohort, we excluded 124 participants of non-European genetic ancestry and performed the associations study again. This did not significantly impact the metabolite associations: of the original 27 putative metabolites, 23 retained statistical significance, while four metabolites lost significance but remained close to the threshold. Index SNP effect sizes and *p* values across both datasets were highly consistent, with a Spearman correlation coefficient of *r*^2^ = 0.9997 and *r*^2^ = 0.9991 (*p* < 0.001), respectively (Figure S5; Table S6). Effect sizes and significance levels from the male-only GWAS were highly concordant with the original analysis, with Pearson’s *r*^2^ = 0.997 for effect sizes and *r*^2^ = 0.996 for *p* values, indicating no meaningful impact of sex stratification on the primary findings (Figure S6; Table S6).

To assess the potential confounding effects of HIV-context-related variables, we conducted a sensitivity analysis including CD4-positive T cell count, HIV-1 viral load, ART regimen, and the duration of the current combination therapy as covariates. Our GWAS results remained highly consistent, with 26/27 putative metabolites retaining statistical significance and one metabolite remaining near the significance threshold (*p* = 5.71 × 10^–11^), supporting the robustness of the associations to HIV-context variable confounding (Figure S7; Table S6). To further explore potential synergistic effects, we performed exploratory SNP × HIV-context interaction analyses; however, none of the interaction terms reached statistical significance after Bonferroni correction (Table S7).

Of the 27 putative metabolites identified with significant genetic associations in our study, 10 have been previously reported in large-scale metabolome-wide association studies (mGWASs)^15^^,^^20^^,^^35^^,^^36^^,^^37^^,^^38^^,^^39^ with eight mQTLs successfully replicated and two showing associations with different loci (Tables S8 and S9).

### Colocalization analysis

We performed colocalization analyses to assess shared genetic associations between mQTLs) and tissue-level eQTLs. Of the 27 putative metabolites with a significant mQTL, 24 shared genetic variants influencing gene expression in at least one tissue (posterior probability PP.H4 ≥ 0.8) (Table S10). These results suggest that some metabolite-associated variants overlap with eQTLs for genes that may regulate the corresponding metabolite levels. Notable examples include variants influencing *FUT2* expression associated with a feature with the formula *C*_12_*H*_22_*O*_10_, which matched with the HMDB entry for 2-O-L-fucopyranosyl-galactose (PP.H4 = 0.95) as well as *NAT8*-linked variants showing associations with putatively annotated N-acetylated metabolites such as N-acetylornithine (*C*_7_*H*_14_*N*_2_*O*_3_, PP.H4 = 0.92) and N-acetylcitrulline (*C*_8_*H*_15_*N*_3_*O*_4_, PP.H4 = 0.98), and *SPTLC3* expression-associated variants linked to ceramide Cer(d18:1/25:0) (*C*_4_*H*_85_*NO*_3_, PP.H4 = 0.99).

### MR

We performed MR analyses to investigate potential causal relationships between the 187 putative metabolites with at least one genome-wide significant genetic variant (*p* < 5 × 10^−8^) identified in the SHCS and a set of clinically relevant continuous biomarkers and binary disease outcomes derived from the UKB and additional external GWAS datasets. Instrument strength appeared adequate across all analyses, with all metabolite-specific *F* statistics exceeding 10 and a minimum value of 30, indicating limited evidence for substantial weak-instrument bias (Table S11).

Using the MR-link-2 method,^56^ which accounts for pleiotropy and reduces false positives compared to non-pleiotropy robust MR methodology, we assessed causal relationships between these metabolites and 18 clinically relevant outcomes derived from UKB summary statistics^45^ and additional external GWAS datasets.^46^^,^^47^^,^^48^^,^^49^^,^^50^^,^^51^^,^^52^^,^^53^^,^^54^ These outcomes included continuous biomarkers and binary disease traits relevant to cardiovascular, kidney, liver, neurodegenerative, and skeletal health, as well as aging-related phenotypes (see section material and methods; Table S12).

This analysis identified four metabolite-trait combinations as putatively causal at a Bonferroni-corrected significance threshold (*p* < 1.48 × 10^−5^). For each significant metabolite-trait association, we examined the MR-link-2 pleiotropy parameter, *σ*, which reflects residual pleiotropic variance. Across all associations passing the multiple-testing-corrected significance threshold, the *σ* estimates were not statistically significant (Table S13), suggesting that the detected causal signals are unlikely to be predominantly driven by horizontal pleiotropy.

**Figure 4:**
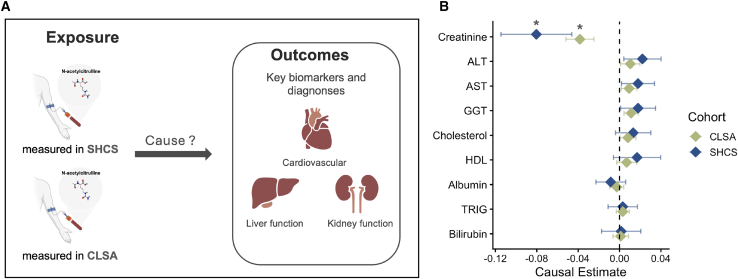
MR analysis of N-acetylcitrulline and clinical outcomes (A) Schematic overview of the analysis strategy. N-acetylcitrulline levels measured in SHCS or CLSA were used as exposures in a two-sample Mendelian randomization (MR) framework. Genetic instruments were tested for causal effects on clinical biomarkers in the UK Biobank (UKB), focusing on cardiovascular, liver, and kidney function outcomes. (B) Forest plot showing MR causal estimates for selected outcomes across SHCS (blue) and CLSA (green) cohorts. Error bars represent 95% confidence intervals. The asterisks (∗) denote associations that reached statistical significance after multiple testing correction. CHOL, cholesterol; GGT, gamma-glutamyl transferase; AST, aspartate aminotransferase; TRIG, triglycerides; ALB, albumin; CREAT, creatinine; HDL, high-density lipoprotein. Figure partially created with BioRender.com.

Reverse MR analyses provided no evidence for a causal effect of the outcomes on these metabolite levels, supporting a unidirectional relationship (Table S14). Notably, the association between N-acetylcitrulline (C_8_H_15_N_3_O_4_) and creatinine (*α* = −0.08, *p* = 4.27 × 10^−6^) was independently replicated in the CLSA (Figure 4). The remaining four significant associations were not measured in the CLSA (Table S15). Among the putatively causal metabolites, chorismate (C_10_H_10_O_6_) and 2-*O*-L-fucopyranosyl-galactose (C_12_H_22_O_10_) were each associated with multiple cardiometabolic traits. 2-*O*-L-fucopyranosyl-galactose exhibited protective effects, with negative causal estimates on cholesterol (*α* = −0.079, *p* = 2.34 × 10^−6^), implicating potential benefits for lipid biomarkers (Figure S9A). In contrast, chorismate was significantly associated with increased levels of cholesterol (*α* = 0.105, *p* = 1.87 × 10^−6^) and triglyceride levels (*α* = 0.180, *p* = 2.36 × 10^−6^), suggesting a potential role in lipid metabolism (Figure S9B).

## Discussion

This genome-wide metabolomics analysis of 1,930 putative plasma metabolites in 1,244 PWH from the SHCS provides insights into metabolic pathways that may contribute to aging-related comorbidities. We identified 27 putative metabolites significantly associated with genetic variants across 12 genomic loci, reinforcing known metabolic pathways and highlighting several associations not previously reported in the comparison studies.

Our study confirms several established genetic determinants of metabolic traits previously reported in general populations, including loci related to lipid metabolism (*FADS1-3*),^15^^,^^37^ amino acid N-acetylation (*NAT8*),^15^^,^^58^^,^^59^^,^^60^ and methyllysine-related metabolites (*PYROXD2*).^61^^,^^62^^,^^63^ These findings underscore that fundamental genetic regulation of metabolic pathways remains robust despite the complexities introduced by HIV infection and ART.

In addition, 10 of the metabolite-genetic variant associations identified in our cohort were also reported in a recently published metabolomics GWAS study in PWH.^64^ Notably, these overlapping associations did not align completely with findings from non-HIV metabolomics GWAS, indicating that certain mQTLs may be more apparent in the context of HIV infection, ART exposure, or related metabolic perturbations. (Table S16).

We identified an intriguing association between the *F11* variant rs4253271 and plasma levels of 5-hydroxytryptophan (5-HTP), a key intermediate in serotonin biosynthesis. The alternative allele T of the associated SNP (rs4253271) is a known expression and protein QTL (eQTL and protein QTL [pQTL]) associated with increased levels of coagulation factor XI. This association was not observed in a larger general-population cohort (Long et al.^36^) but was observed in an HIV population study (2000HIV cohort study)^64^ (Table S16), raising the possibility that this association may be more readily detectable in PWH cohorts. It has been reported that HIV infection perturbs tryptophan metabolism via immune activation and induction of the kynurenine pathway,^65^ which might explain why this genotype-metabolite association has not been detected in other settings.

Our results also expand the scope of known metabolic associations with the *PYROXD2* gene to include additional metabolites detected in our analysis; i.e., propionylcholine (*C*_8_*H*_18_*NO*_2_), (E)-4-(trimethylammonio)but-2-enoate (also known as crotonic acid betaine, *C*_7_*H*_13_*NO*_2_), and syringoylglycerol (*C*_11_*H*_16_*O*_6_). Notably, both propionylcholine and crotonic acid betaine, alongside previously identified methyl-lysine metabolites, contain methylated amino groups and localize to the same genetic locus as trimethylamine (TMA), a well-established *PYROXD2*-associated metabolite.^66^ TMA is a microbial precursor of trimethylamine-N-oxide (TMAO), a microbial-host co-metabolite with strong links to cardiovascular disease risk.^67^^,^^68^ This combination of structural similarity and genetic colocalization suggests a broader role for *PYROXD2* in regulating the TMA metabolic axis, highlighting its potential involvement in cardiovascular and microbiome-related health outcomes.

Further supporting the role of microbial metabolites in cardiovascular health, a significant causal relationship was found by MR analysis between an elevated metabolite concentration with the formula *C*_10_*H*_10_*O*_6_, matching the HMDB entry for chorismate, and increased circulating cholesterol and triglycerides levels. Chorismate is an intermediate of the bacterial shikimate pathway, absent from human metabolism.^69^ While our data do not directly measure gut permeability or microbiome composition, the detection of chorismate in plasma is consistent with increased microbial translocation previously described in PWH.^70^ Genetic associations between chorismate and lipid traits show visual overlap at the *NAT2* locus region (Figure S8). *NAT2* encodes a xenobiotic-metabolizing enzyme predominantly expressed in the liver and intestines,^71^ implicating host genetic variation in detoxification capacity as a potential modulator of the cardiometabolic effects of microbial-derived metabolites such as chorismate.

MR provided evidence consistent with putative causal relationships between circulating metabolites and clinical biomarkers, including an inverse association between genetically predicted N-acetylcitrulline levels and serum creatinine, observed consistently across both the SHCS and CLSA cohorts. Genetically elevated N-acetylcitrulline levels were associated with significantly reduced creatinine concentrations, suggesting a protective effect on kidney function. In our GWAS, the lead SNP for N-acetylcitrulline was rs10182082, which is in LD (*R*^2^ = 0.98) with rs13538, a missense variant (p.Phe143Ser) in the *NAT8* gene. This variant alters the acetyl-coenzyme A (CoA) binding site^59^ and has been shown to reduce NAT8 protein expression. Importantly, rs13538 has been previously associated with lower estimated glomerular filtration rate (eGFR) in public GWASs.^72^ In our data, the alternative allele (A) of rs13538 was significantly associated with lower N-acetylcitrulline levels (*β* = −0.51, *p* = 1.48 × 10^–28^), and, through MR, we show that lower N-acetylcitrulline levels causally lead to higher serum creatinine concentration, a surrogate for impaired kidney function (Figure 4). Taken together, these findings support a model in which *NAT8* genetic variation modulates the homeostasis of N-acetylated metabolites, thereby influencing kidney function. They underscore the potential role of altered N-acetylation dynamics as a mechanistic link between metabolic regulation and renal health.

The current study has several limitations. While we detected 1,930 metabolic features with high confidence in their molecular formulas, the precise structural identities of many remain uncertain. These should therefore be regarded as putative metabolites rather than fully validated compounds. Second, our analyses were conducted within a single HIV cohort, which may introduce selection bias and limit the generalizability of the findings to broader or more diverse populations. Opportunities for external validation were further constrained by differences in metabolomic platforms and by the limited number of individuals living with HIV in large population-based resources. In addition, our two-sample MR analyses relied on a general-population-outcome GWAS, which assumes that the underlying genetic effects are sufficiently transferable between PWH and non-HIV populations. For metabolites not measured in external non-HIV datasets, this assumption could not be directly assessed, and these associations should therefore be interpreted cautiously as putative causal associations rather than definitive evidence of HIV-specific causal effects. Nevertheless, some PWH-derived signals may reflect genetic effects that become more apparent in the context of HIV infection, ART exposure, or related xenobiotic and metabolic perturbations. A further consideration is that, after LD clumping, many metabolite exposures were supported by only one or a few independent genetic instruments. As a result, conventional multi-instrument sensitivity analyses were not applicable. Although MR-link-2 provides a framework for estimating causal effects and residual pleiotropy in single-region settings, the limited number of independent instruments restricted the range of complementary MR sensitivity analyses that could be performed. The integration of findings with external studies was further challenged by inconsistencies in metabolite nomenclature and identification conventions, particularly when different detection platforms were used. Our harmonization approach, although systematic, did not enable unambiguous cross-study comparisons for all metabolites. The implementation of standardized, community-adopted frameworks for metabolite identification and annotation would substantially improve the reproducibility and interpretability of metabolomics findings across diverse cohorts. Finally, we did not collect detailed dietary or lifestyle information. Although samples were obtained after an overnight fast, residual confounding from longer-term behaviors may remain.

Future studies incorporating larger sample sizes, ethnically diverse populations, and matched control groups will be critical to validate and extend the generalizability of these results. Exploring the mechanistic pathways through targeted functional studies could enhance our understanding of gene-microbiome-metabolite interactions in the context of HIV infection and aging. Moreover, longitudinal analyses could elucidate the clinical impact of these genetic and metabolic findings on the trajectory of co-morbid diseases in PWH. Finally, integrating established biomarkers of aging such as epigenetic clocks and telomere length could provide more precise measures of biological aging and help disentangle the contributions of HIV, host genetics, and metabolic alterations to accelerated aging processes in PWH.

To our knowledge, this study represents one of the largest untargeted metabolome-wide GWAS analysis conducted in PWH to date. By leveraging untargeted metabolomics, we identified genetic-metabolic associations in PWH, including signals that overlap with general-population metabolic regulation and signals that may be more apparent in the context of HIV infection, ART exposure, or related metabolic perturbations. These findings generate hypotheses for future mechanistic studies and may ultimately help inform precision approaches to metabolic and comorbidity-related conditions in PWH.

## Data and code availability

The code used for mapping metabolites identifiers to HMDB ID can be downloaded at https://github.com/mariamaitoumelloul/MetaboMapper.

## Consortia

SHCS.

Abela I.A., Aebi-Popp K, Anagnostopoulos A., Battegay M., Bernasconi E., Braun D.L., Bucher H.C., Calmy A., Cavassini M. (Chairman of the Clinical and Laboratory Committee), Ciuffi A., Dollenmaier G., Egger M., Elzi L., Fehr J.S., Fellay J., Frigerio Malossa S., Furrer H., Fux C.A., Günthard H.F., Hachfeld A., Haerry D.H.U. (deputy of “Positive Council”), Hasse B., Hirsch H.H., Hoffmann M., Hösli I., Huber M., Jackson-Perry D. (patient representatives), Kahlert C.R. (Chairman of the Mother & Child Substudy), Keiser O., Klimkait T., Kouyos R.D., Kovari H., Kusejko K. (Head of Data Centre), Labhardt N.D., Leuzinger K., Martinez de Tejada B., Marzolini C., Metzner K.J., Müller N., Nemeth J., Nicca D., Notter J., Paioni P., Pantaleo G., Perreau M., Rauch A. (President of the SHCS), Salazar-Vizcaya L.P., Schmid P., Segeral O., Speck R.F., Stöckle M., Tarr P.E., Trkola A., Wandeler G. (Chairman of the Scientific Board), Weisser M., Yerly S.

## Acknowledgments

This study has been financed within the framework of the Swiss HIV Cohort Study, supported by the 10.13039/501100001711Swiss National Science Foundation (grant #33FI-0_229621), by SHCS project #877, and by the SHCS research foundation. The data are gathered by the Five Swiss University Hospitals, two Cantonal Hospitals, 15 affiliated hospitals, and 36 private physicians (listed in http://www.shcs.ch/180-health-care-providers).

The authors acknowledge the effort and commitment of SHCS participants, investigators, study nurses, laboratory personnel, and administrative assistance by the SHCS coordination and data center.

## Author contributions

Study design, M.A.O., A.v.d.G., J.F, and P.E.T.; data management and participant selection, M.L. and R.D.K.; data acquisition, I.C.S., A.C.., and P.E.T.; data analysis, M.A.O., A.v.d.G., and S.T.; drafting of the manuscript, M.A.O. and J.F.; critical review and revision of the manuscript, all authors.

## Declaration of interests

P.E.T.’s institution reports grants, advisory fees, or educational fees from Gilead, ViiV, MSD, and Daiichi-Sankyo, outside the submitted work. M.S.’s institution received payments for advisory board activities from Gilead, MSD, ViiV, and Moderna and received grants for conference participation from Gilead and MSD.

## Supplemental information

Document S1. Figures S1–S9

Table S1. Information on metabolites detected through untargeted metabolomics in the current study

Table S2. Associations of individual metabolites with demographic variables, including age, sex assigned at birth, smoking, and genetic ancestry, together with effect sizes and *p* values

Table S3. SNP-based heritability estimates

Data S1. Tables S4 and S5

Table S6. Genome-wide significant SNP-metabolite associations in the full cohort, including locus information, alleles, effect sizes, and *p* values The table also includes results from analyses restricted to the European ancestry subset and analyses adjusted for HIV-context covariates.

Table S7. Results from the synergistic interaction analyses between metabolites and HIV-context variables, reporting all SNP × HIV-variable interaction terms that exceeded the prespecified suggestive significance threshold

Data S2. Tables S8 and S9

Table S10. eQTL analyses linking GWAS loci to tissue-specific gene expression

Table S11. Assessment of genetic instrumental variable strength for metabolites included in MR analyses, reporting the number of instruments, variance explained, and corresponding *F* statistics for each metabolite

Table S12. GWAS outcomes from external databases tested in two-sample MR

Table S13. MR results for SHCS metabolite and aging-related comorbidities and key biomarkers of organ function from external cohorts, reporting effect estimates, confidence intervals, significance, and pleiotropy coefficient

Table S14. The results of reverse MR analyses

Table S15. Corresponding MR results for CLSA metabolite exposures and aging-related comorbidities and key biomarkers of organ function from external cohorts, reporting effect estimates, confidence intervals, significance, and pleiotropy coefficient

Table S16. Metabolite-QTL associations identified across different studies For each metabolite, including molecular formula and putative annotation, the table indicates whether an association was detected in the 2000HIV study, in the SHCS cohort, or in non-HIV studies.

Document S2. Article plus supplemental information

## References

[1] World Health Organization (2024). https://www.who.int/news-room/fact-sheets/detail/hiv-aids.

[2] Freiberg M.S., Chang C.C.H., Kuller L.H., Skanderson M., Lowy E., Kraemer K.L., Butt A.A., Bidwell Goetz M., Leaf D., Oursler K.A. (2013). “HIV infection and the risk of acute myocardial infarction”. JAMA Intern. Med..

[3] Shah A.S.V., Stelzle D., Lee K.K., Beck E.J., Alam S., Clifford S., Longenecker C.T., Strachan F., Bagchi S., Whiteley W. (2018). “Global Burden of Atherosclerotic Cardiovascular Disease in People Living With HIV: Systematic Review and Meta-Analysis”. Circulation.

[4] Feinstein M.J., Hsue P.Y., Benjamin L.A., Bloomfield G.S., Currier J.S., Freiberg M.S., Grinspoon S.K., Levin J., Longenecker C.T., Post W.S. (2019). “Characteristics, Prevention, and Management of Cardiovascular Disease in People Living With HIV: A Scientific Statement From the American Heart Association”. Circulation.

[5] Heron J.E., Bagnis C.I., Gracey D.M. (2020). “Contemporary issues and new challenges in chronic kidney disease amongst people living with HIV”. AIDS Res. Ther..

[6] Sterling R.K., Chiu S., Snider K., Nixon D. (2008-05). “Theprevalence and risk factors for abnormal liver enzymes in HIV positive patients without hepatitis b or c coinfections”. Dig. Dis. Sci..

[7] Michel M., Labenz C., Armandi A., Kaps L., Kremer W.M., Galle P.R., Grimm D., Sprinzl M., Schattenberg J.M. (2023-06-06). “Metabolic dysfunction-associated fatty liver disease in people living with HIV”. Sci. Rep..

[8] Bar N., Korem T., Weissbrod O., Zeevi D., Rothschild D., Leviatan S., Kosower N., Lotan-Pompan M., Weinberger A., Le Roy C.I. (2020). “A reference map of potential determinants for the human serum metabolome”. Nature.

[9] Pietzner M., Stewart I.D., Raffler J., Khaw K.T., Michelotti G.A., Kastenmüller G., Wareham N.J., Langenberg C. (2021). “Plasma metabolites to profile pathways in noncommunicable disease multimorbidity”. Nat. Med..

[10] Clish C.B. (2015). “Metabolomics: An emerging but powerful tool for precision medicine”. Cold Spring Harb. Mol. Case Stud..

[11] Lu L., Yang Y., Yang Z., Wu Y., Liu X., Li X., Chen L., Han Y., Song X., Kong Z. (2023). “Altered plasma metabolites and inflammatory networks in HIV-1 infected patients with different immunological responses after long-term antiretroviral therapy”. Front. Immunol..

[12] Virseda-Berdices A., Martín-Escolano R., Berenguer J., González-García J., Brochado-Kith O., Rojo D., Fernández-Rodríguez A., Pérez-Latorre L., Hontañón V., Barbas C. (2024). “Plasma metabolomic profile is near-normal in people with HIV on long-term suppressive antiretroviral therapy”. Front. Cell. Infect. Microbiol..

[13] Labarile M. (2025). “Untargeted Metabolite Profile Associations with Body Mass Index, Waist-Hip Ratio, and Antiretroviral Therapy in >1300 People with HIV: The Swiss HIV Cohort Study”. J. Infect. Dis..

[14] Gieger C., Geistlinger L., Altmaier E., Hrabé de Angelis M., Kronenberg F., Meitinger T., Mewes H.W., Wichmann H.E., Weinberger K.M., Adamski J. (2008). “Genetics meets metabolomics: A genome-wide association study of metabolite profiles in human serum”. PLoS Genet..

[15] Chen Y., Lu T., Pettersson-Kymmer U., Stewart I.D., Butler-Laporte G., Nakanishi T., Cerani A., Liang K.Y.H., Yoshiji S., Willett J.D.S. (2023). “Genomic atlas of the plasma metabolome prioritizes metabolites implicated in human diseases”. Nat. Genet..

[16] Chu X., Jaeger M., Beumer J., Bakker O.B., Aguirre-Gamboa R., Oosting M., Smeekens S.P., Moorlag S., Mourits V.P., Koeken V.A.C.M. (2021). “Integration of metabolomics, genomics, and immune phenotypes reveals the causal roles of metabolites in disease”. Genome Biol..

[17] Ge A., Sun Y., Kiker T., Zhou Y., Ye K. (2023). “A metabolome-wide mendelian randomization study prioritizes potential causal circulating metabolites for multiple sclerosis”. J. Neuroimmunol..

[18] Zeng Y., Liu H., Pei Z., Li R., Liu Z., Liao C. (2024). “Evaluation of the causal effects of blood metabolites on irritable bowel syndrome: Mendelian randomization”. BMC Gastroenterol..

[19] Schlosser P., Li Y., Sekula P., Raffler J., Grundner-Culemann F., Pietzner M., Cheng Y., Wuttke M., Steinbrenner I., Schultheiss U.T. (2020). “Genetic studies of urinary metabolites illuminate mechanisms of detoxification and excretion in humans”. Nat. Genet..

[20] Schlosser P., Scherer N., Grundner-Culemann F., Monteiro-Martins S., Haug S., Steinbrenner I., Uluvar B., Wuttke M., Cheng Y., Ekici A.B. (2023). “Genetic studies of paired metabolomes reveal enzymatic and transport processes at the interface of plasma and urine”. Nat. Genet..

[21] Scherrer A.U., Traytel A., Braun D.L., Calmy A., Battegay M., Cavassini M., Furrer H., Schmid P., Bernasconi E., Stoeckle M. (2022). “Cohort profile update: The swiss HIV cohort study (SHCS)”. Int. J. Epidemiol..

[22] The 1000 Genomes Project Consortium (2010). “A map of human genome variation from population-scale sequencing”. Nature.

[23] The 1000 Genomes Project Consortium (2012). “An integrated map of genetic variation from 1,092 human genomes”. Nature.

[24] Purcell S., Neale B., Todd-Brown K., Thomas L., Ferreira M.A.R., Bender D., Maller J., Sklar P., de Bakker P.I.W., Daly M.J., Sham P.C. (2007). “PLINK: A tool set for whole-genome association and population-based linkage analyses”. Am. J. Hum. Genet..

[25] Fuhrer T., Zamboni N. (2015). “High-throughput discovery metabolomics”. Curr. Opin. Biotechnol..

[26] Wishart D.S., Feunang Y.D., Marcu A., Guo A.C., Liang K., Vázquez-Fresno R., Sajed T., Johnson D., Li C., Karu N. (2018). “HMDB 4.0: The human metabolome database for 2018”. Nucleic Acids Res..

[27] Wishart D.S., Guo A., Oler E., Wang F., Anjum A., Peters H., Dizon R., Sayeeda Z., Tian S., Lee B. (2022). “HMDB 5.0: The human metabolome database for 2022”. Nucleic Acids Res..

[28] Lopez-Ibañez J., Pazos F., Chagoyen M. (2023). “MBROLE3: Improved functional enrichment of chemical compounds for metabolomics data analysis”. Nucleic Acids Res..

[29] Yang J., Lee S.H., Wray N.R., Goddard M.E., Visscher P.M. (2016). “GCTA-GREML accounts for linkage disequilibrium when estimating genetic variance from genome-wide SNPs”. Proc. Natl. Acad. Sci. USA.

[30] Visscher P.M., Hemani G., Vinkhuyzen A.A.E., Chen G.B., Lee S.H., Wray N.R., Goddard M.E., Yang J. (2014). “Statistical power to detect genetic (co)variance of complex traits using SNP data in unrelated samples”. PLoS Genet..

[31] Mbatchou J., Barnard L., Backman J., Marcketta A., Kosmicki J.A., Ziyatdinov A., Benner C., O’Dushlaine C., Barber M., Boutkov B. (2021). “Computationally efficient whole-genome regression for quantitative and binary traits”. Nat. Genet..

[32] Yang J., Lee S.H., Goddard M.E., Visscher P.M. (2011). “GCTA: A tool for genome-wide complex trait analysis”. Am. J. Hum. Genet..

[33] Yang J., Ferreira T., Morris A.P., Medland S.E., Madden P.A.F., Heath A.C., Martin N.G., Montgomery G.W., Weedon M.N., Loos R.J. (2012). “Conditional and joint multiple-SNP analysis of GWAS summary statistics identifies additional variants influencing complex traits”. Nat. Genet..

[34] Watanabe K., Taskesen E., van Bochoven A., Posthuma D. (2017). “Functional mapping and annotation of genetic associations with FUMA”. Nat. Commun..

[35] Shin S.-Y., Fauman E.B., Petersen A.K., Krumsiek J., Santos R., Huang J., Arnold M., Erte I., Forgetta V., Yang T.P. (2014). “An atlas of genetic influences on human blood metabolites”. Nat. Genet..

[36] Long T., Hicks M., Yu H.C., Biggs W.H., Kirkness E.F., Menni C., Zierer J., Small K.S., Mangino M., Messier H. (2017). “Whole-genome sequencing identifies common-to-rare variants associated with human blood metabolites”. Nat. Genet..

[37] Lotta L.A., Pietzner M., Stewart I.D., Wittemans L.B.L., Li C., Bonelli R., Raffler J., Biggs E.K., Oliver-Williams C., Auyeung V.P.W. (2021). “A cross-platform approach identifies genetic regulators of human metabolism and health”. Nat. Genet..

[38] Hysi P.G., Mangino M., Christofidou P., Falchi M., Karoly E.D., Mohney R.P., Valdes A.M., Spector T.D., Menni C. (2022). “Metabolome genome-wide association study identifies 74 novel genomic regions influencing plasma metabolites levels”. Metabolites.

[39] Yin X., Bose D., Kwon A., Hanks S.C., Jackson A.U., Stringham H.M., Welch R., Oravilahti A., Fernandes Silva L., Locke A.E. (2022). “Integrating transcriptomics, metabolomics, and GWAS helps reveal molecular mechanisms for metabolite levels and disease risk”. Am. J. Hum. Genet..

[40] Kerimov N., Tambets R., Hayhurst J.D., Rahu I., Kolberg P., Raudvere U., Kuzmin I., Chowdhary A., Vija A., Teras H.J. (2023). “eQTL catalogue 2023: New datasets, x chromosome QTLs, and improved detection and visualisation of transcript-level QTLs”. PLoS Genet..

[41] GTEx Consortium (2017). “Genetic effects on gene expression across human tissues”. Nature.

[42] Verdi S., Abbasian G., Bowyer R.C.E., Lachance G., Yarand D., Christofidou P., Mangino M., Menni C., Bell J.T., Falchi M. (2019). “TwinsUK: The UK adult twin registry update”. Twin Res. Hum. Genet..

[43] Wallace C., Giambartolomei C. (2012).

[44] Sanderson E., Glymour M.M., Holmes M.V., Kang H., Morrison J., Munafò M.R., Palmer T., Schooling C.M., Wallace C., Zhao Q., Davey Smith G. (2022). “Mendelian randomization”. Nat. Rev. Methods Primers.

[45] Karczewski K.J., Gupta R., Kanai M. (2025). Pan-UK Biobank genome-wide association analyses enhance discovery and resolution of ancestry-enriched effects. Nat. Genet..

[46] Morris J.A., Kemp J.P., Youlten S.E., Laurent L., Logan J.G., Chai R.C., Vulpescu N.A., Forgetta V., Kleinman A., Mohanty S.T. (2019). “An atlas of genetic influences on osteoporosis in humans and mice”. Nat. Genet..

[47] Codd V., Wang Q., Allara E., Musicha C., Kaptoge S., Stoma S., Jiang T., Hamby S.E., Braund P.S., Bountziouka V. (2021). “Polygenic basis and biomedical consequences of telomere length variation”. Nat. Genet..

[48] Suzuki K., Hatzikotoulas K., Southam L., Taylor H.J., Yin X., Lorenz K.M., Mandla R., Huerta-Chagoya A., Melloni G.E.M., Kanoni S. (2024). “Genetic drivers of heterogeneity in type 2 diabetes pathophysiology”. Nature.

[49] Wuttke M., Li Y., Li M., Sieber K.B., Feitosa M.F., Gorski M., Tin A., Wang L., Chu A.Y., Hoppmann A. (2019). “A catalog of genetic loci associated with kidney function from analyses of a million individuals”. Nat. Genet..

[50] Mishra A., Malik R., Hachiya T., Jürgenson T., Namba S., Posner D.C., Kamanu F.K., Koido M., Le Grand Q., Shi M. (2022). “Stroke genetics informs drug discovery and risk prediction across ancestries”. Nature.

[51] van der Harst P., Verweij N. (2018). “Identification of 64 Novel Genetic Loci Provides an Expanded View on the Genetic Architecture of Coronary Artery Disease”. Circ. Res..

[52] Wightman D.P., Jansen I.E., Savage J.E., Shadrin A.A., Bahrami S., Holland D., Rongve A., Børte S., Winsvold B.S., Drange O.K. (2021). “A genome-wide association study with 1,126,563 individuals identifies new risk loci for Alzheimer’s disease”. Nat. Genet..

[53] Nalls M.A., Blauwendraat C., Vallerga C.L., Heilbron K., Bandres-Ciga S., Chang D., Tan M., Kia D.A., Noyce A.J., Xue A. (2019). “Identification of novel risk loci, causal insights, and heritable risk for Parkinson’s disease: A meta-genome wide association study”. Lancet Neurol..

[54] Ghouse J., Sveinbjörnsson G., Vujkovic M., Seidelin A.S., Gellert-Kristensen H., Ahlberg G., Tragante V., Rand S.A., Brancale J., Vilarinho S. (2024). “Integrative common and rare variant analyses provide insights into the genetic architecture of liver cirrhosis”. Nat. Genet..

[55] Burgess S., Thompson S.G., CRP CHD Genetics Collaboration (2011). “Avoiding bias from weak instruments in Mendelian randomization studies”. Int. J. Epidemiol..

[56] van der Graaf A., Warmerdam R., Auwerx C. (2025). MR-link-2: pleiotropy robust cis Mendelian randomization validated in three independent reference datasets of causality. Nat. Commun..

[57] Manini P. (2003). “[Urinary excretion of 4-vinyl phenol after experimental and occupational exposure to styrene]”. G Ital Med Lav Ergon.

[58] Suhre K., Shin S.Y., Petersen A.K., Mohney R.P., Meredith D., Wägele B., Altmaier E., Deloukas P., Erdmann J., Grundberg E. (2011). “Human metabolic individuality in biomedical and pharmaceutical research”. Nature.

[59] Luo S., Surapaneni A., Zheng Z., Rhee E.P., Coresh J., Hung A.M., Nadkarni G.N., Yu B., Boerwinkle E., Tin A. (2021). “NAT8 variants, n-acetylated amino acids, and progression of CKD”. Clin. J. Am. Soc. Nephrol..

[60] Luo S., Feofanova E.V., Tin A., Tung S., Rhee E.P., Coresh J., Arking D.E., Surapaneni A., Schlosser P., Li Y. (2021). “Genome-wide association study of serum metabolites in the african american study of kidney disease and hypertension”. Kidney Int..

[61] Schlosser P., Hackenberg M., Monteiro-Martins S., Haug S., Kottgen A. (2024). “Network analysis of paired plasma-urine metabolomes reveals genetic determinants of metabolite clusters: TH-OR20”. J. Am. Soc. Nephrol..

[62] Lee I.-H., Smith M.R., Yazdani A., Sandhu S., Walker D.I., Mandl K.D., Jones D.P., Kong S.W. (2022). “Comprehensive characterization of putative genetic influences on plasma metabolome in a pediatric cohort”. Hum. Genomics.

[63] Moore A., Busch M.P., Dziewulska K., Francis R.O., Hod E.A., Zimring J.C., D’Alessandro A., Page G.P. (2022). “Genome-wide metabolite quantitative trait loci analysis (mQTL) in red blood cells from volunteer blood donors”. J. Biol. Chem..

[64] Botey-Bataller J., van Unen N., Blaauw M., Vos W.A.J.W., van Eekeren L., Vadaq N., Matzaraki V., Verbon A., Groenendijk A.L., dos Santos J.C. (2025). “Genetic and molecular landscape of comorbidities in people living with HIV”. Nat. Med..

[65] Jenabian M.-A., Patel M., Kema I., Kanagaratham C., Radzioch D., Thébault P., Lapointe R., Tremblay C., Gilmore N., Ancuta P., Routy J.P. (2013). “Distinct tryptophan catabolism and Th17/treg balance in HIV progressors and elite controllers”. PLoS One.

[66] Van Bergen N.J., Hock D.H., Spencer L., Massey S., Stait T., Stark Z., Lunke S., Roesley A., Peters H., Lee J.Y. (2022). Biallelic variants in PYROXD2 cause a severe infantile metabolic disorder affecting mitochondrial function. Int. J. Mol. Sci..

[67] Wang Z., Klipfell E., Bennett B.J., Koeth R., Levison B.S., DuGar B., Feldstein A.E., Britt E.B., Fu X., Chung Y.M. (2011). “Gut flora metabolism of phosphatidylcholine promotes cardiovascular disease”. Nature.

[68] Ding S., Xue J., Zhang Q., Zheng L. (2022). “Trimethylamine-n-oxide is an important target for heart and brain diseases”. Med Rev.

[69] Dosselaere F., Vanderleyden J. (2001). “A metabolic node in action: Chorismate-utilizing enzymes in microorganisms”. Crit. Rev. Microbiol..

[70] Douek D. (2007). “HIV disease progression: Immune activation, microbes, and a leaky gut”. Top. HIV Med..

[71] Husain A., Zhang X., Doll M.A., States J.C., Barker D.F., Hein D.W. (2007). “Identification of n-acetyltransferase 2 (NAT2) transcription start sites and quantitation of NAT2-specific mRNA in human tissues”. Drug Metab. Dispos..

[72] Pattaro C., Teumer A., Gorski M., Chu A.Y., Li M., Mijatovic V., Garnaas M., Tin A., Sorice R., Li Y. (2016). Genetic associations at 53 loci highlight cell types and biological pathways relevant for kidney function. Nat. Commun..

