# Supplementary material for: Genome-wide association study of untargeted plasma metabolomic profiles identifies host genetic regulation in people with HIV
Source: HGG Adv. 2026 Jun 17;7(4):100635. doi: 10.1016/j.xhgg.2026.100635 (PMC13355647; doi:10.1016/j.xhgg.2026.100635)
Supplement: Document S1. Figures S1–S9 [file mmc1.pdf]

HGGA, Volume 7

Supplemental information

Genome-wide association study of untargeted plasma metabolomic profiles identifies host genetic regulation in people with HIV

Mariam Ait Oumelloul, Adriaan van der Graaf, Simon Tang, Christian W. Thorball, Marco Labarile, Ali Saadat, Valeriia Timonina, Isabella C. Schöpf, Gilles Wandeler, Johannes Nemeth, Matthias Cavassini, Alexandra Calmy, Patrick Schmid, Marcel Stöckle, Luigia Elzi, Nicola Zamboni, Roger D. Kouyos, Philip E. Tarr, Jacques Fellay, and Swiss HIV Cohort Study

Supplementary figures

Figure S1. Genetic PCA of SHCS merged with 1000 Genomes Project populations reference samples (AFR: African; AMR: Admixed American; EAS: East Asian; EUR: European)

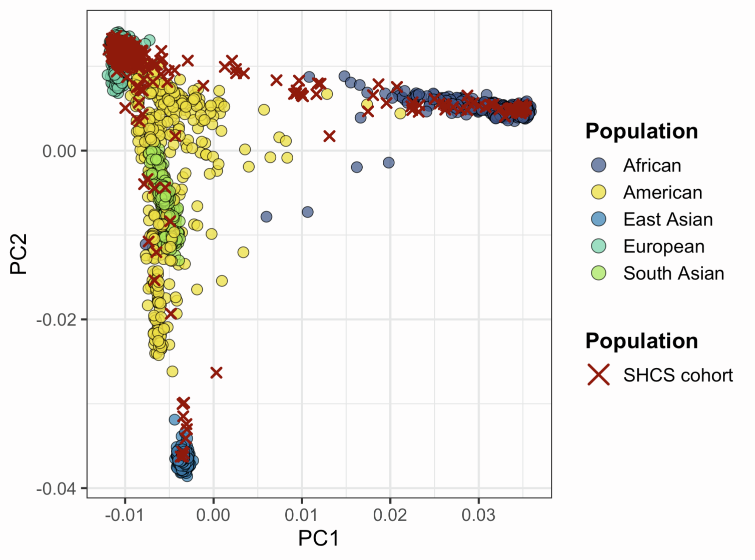

Figure S2. Analytical workflow for two-sample Mendelian randomization analyses. Circulating plasma metabolites with genome-wide significant genetic associations (P $<5\times10^{-8}$) identified in the Swiss HIV Cohort Study (SHCS) were selected as exposures. Genetic instruments were obtained from SHCS GWAS and, in a complementary analysis, from the Canadian Longitudinal Study on Aging (CLSA) for metabolites replicated across both cohorts.Outcomes of interest were obtained from UK Biobank (UKB) GWAS summary statistics. Two-sample Mendelian randomization analyses were conducted to estimate causal effects while accounting for horizontal pleiotropy using MR-link-2. Statistical significance was assessed using Bonferroni-corrected thresholds for metabolite–trait tests, and results obtained using SHCS- and CLSA-derived instruments were compared for consistency.

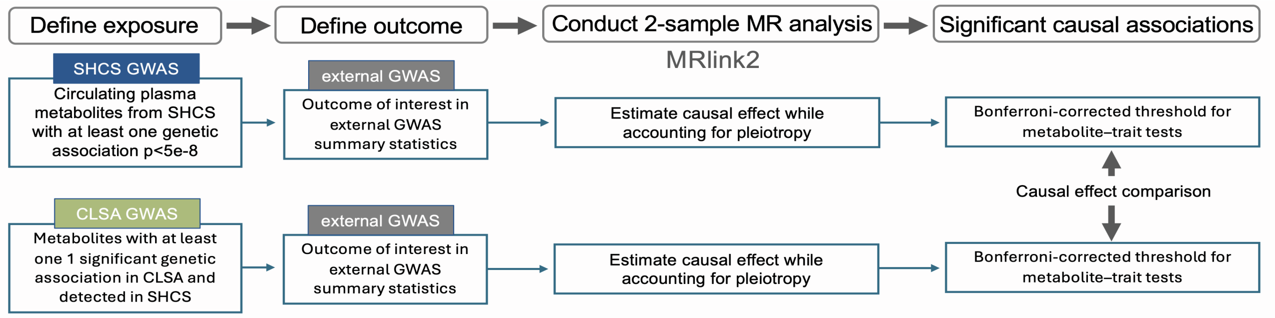

Figure S3. Hierarchical heat map showing pairwise correlations between metabolites. Metabolites are separated by and hierarchically clustered within the 5 super pathways (shown on left) annotated using public databases. Metabolites within the lipid super pathway were also associated to different lipid classes using the chemical taxonomy information from the HMDB (v5.0). The black bands for age, sex, ethnicity (european or others), and smoking indicate statistically significant p values ($p < 0.05$), after accounting for false discovery rate from multiple testing.

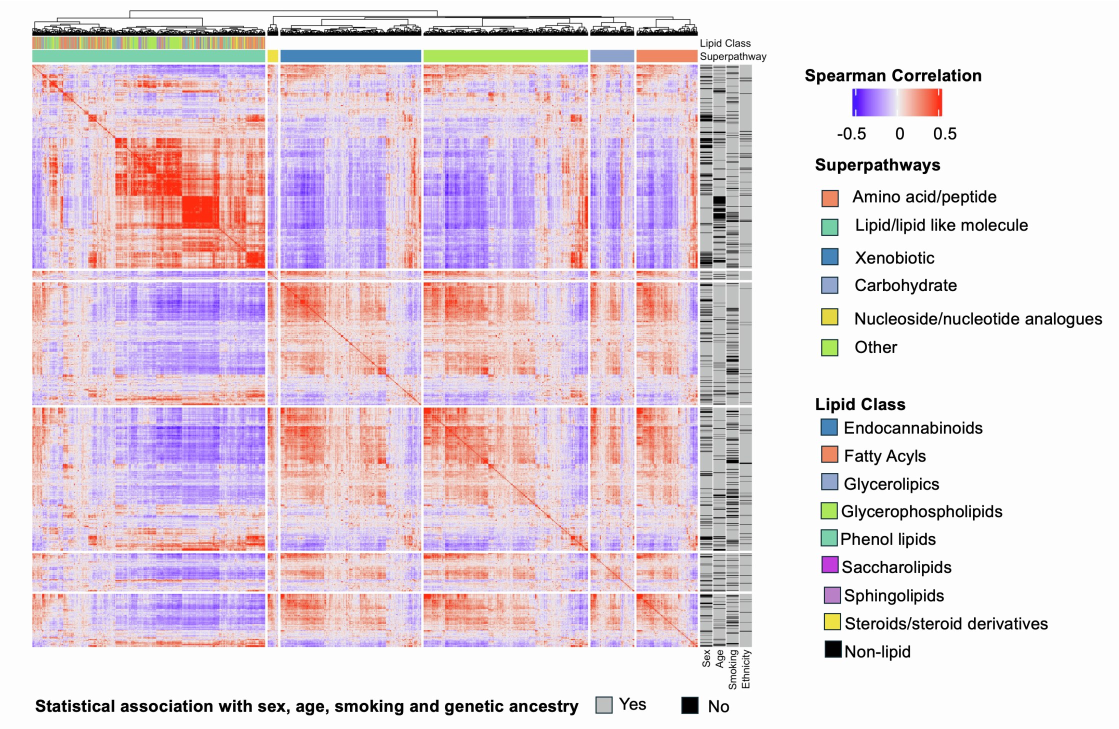

Figure S4. Heritability estimates of significantly heritable metabolites across superpathways. Violin plots show the distribution of SNP-based heritability (h^2) estimates for metabolites (power = 1) with statistically significant heritability (FDR < 0.05), stratified by superpathway. Each dot represents an individual metabolite, and the black dot indicates the median heritability within each category. Superpathways include lipid, xenobiotic, amino acid/peptide, carbohydrate, nucleoside/nucleotide and other.

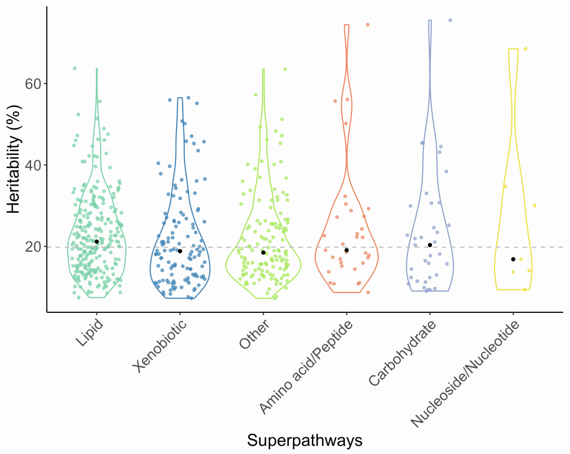

Figure S5. Comparison of genetic associations between the full cohort and European ancestry subgroup. (a) Scatter plot comparing effect sizes (β coefficients) of metabolite-associated variants between the full cohort (x-axis) and the European ancestry subgroup (y-axis). Each dot represents a lead variant-metabolite association from the full cohort analysis. (b) Scatter plot of association significance, comparing –log10(P) for the same variants between the full cohort (x-axis) and European subgroup (y-axis). Dotted red lines indicate linear regression fit. Dashed grey lines mark nominal significance thresholds.

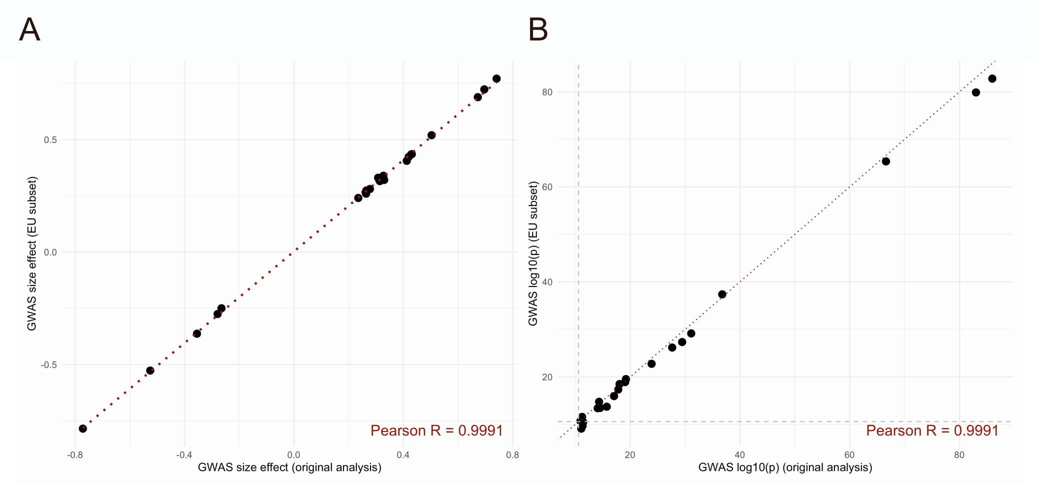

Figure S6. Comparison of genetic associations between the full cohort and male subgroup (based on sex assigned at birth). (A) Scatter plot comparing effect sizes (β coefficients) of metabolite-associated variants between the full cohort (x-axis) and sub-cohort including individuals with male sex assigned at birth only (y-axis). Each dot represents a lead variant-metabolite association from the full cohort analysis. (B) Scatter plot of association significance, comparing –log10(P) for the same variants between the full cohort (x-axis) and and sub-cohort including individuals with male sex assigned at birth only (y-axis). Dotted red lines indicate linear regression fit. Dashed grey lines mark nominal significance thresholds.

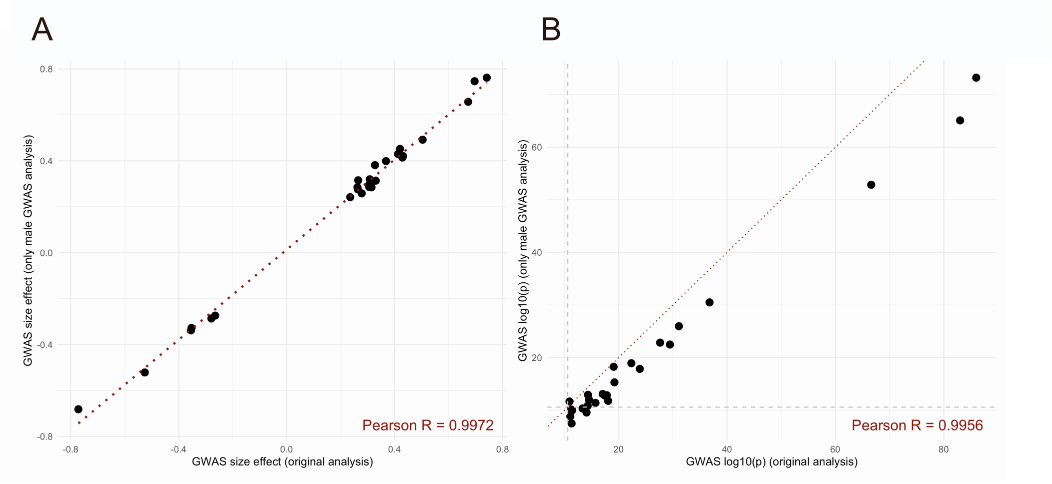

Figure S7. Comparison of genetic associations between the original analysis and analysis when we add HIV-context variables as covariates (CD4 positive T cell count, viral RNA load, ART regiment and duration). (A) Scatter plot comparing effect sizes (β coefficients) of metabolite-associated variants between the original analysis (x-axis) and the sensitivity analysis (y-axis). Each dot represents a lead variant-metabolite association from the original analysis.(B) Scatter plot of association significance, comparing –log10(P) for the same variants between the original analysis (x-axis) and sensitivity analysis (y-axis). Dotted red lines indicate linear regression fit. Dashed grey lines mark nominal significance thresholds.

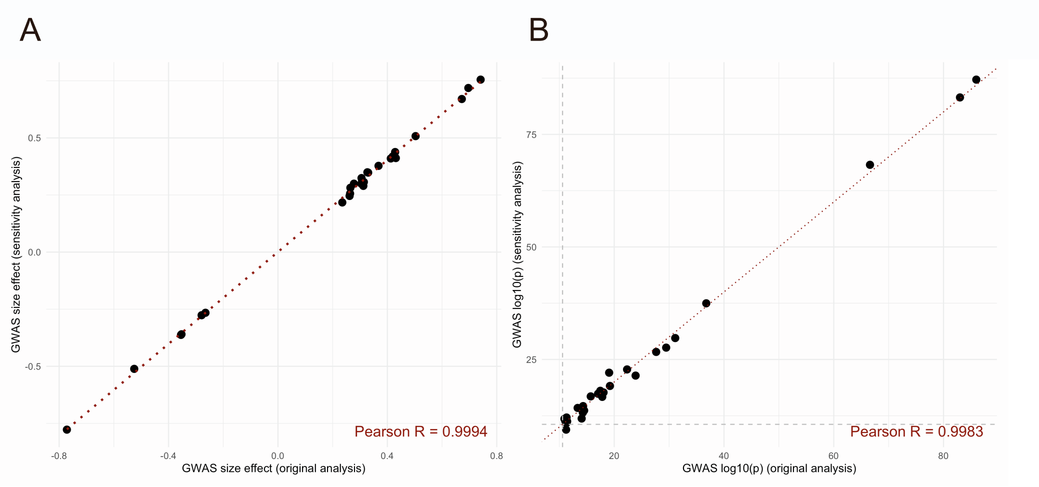

Figure S8. Colocalization of genetic signals associated with chorismate levels in SHCS and lipid traits in the UK Biobank. Regional association plots for the locus on chromosome 8 showing shared genetic architecture between chorismate levels measured in the Swiss HIV Cohort Study (SHCS) and lipid traits (cholesterol and triglyceride) measured in the UK Biobank (UKB). (A) Upper panel: Association of SNPs with chorismate levels in SHCS. Lower panel: Association with total cholesterol in UKB. (B) Upper panel: Chorismate association signal in SHCS. Lower panel: Association with triglyceride levels in UKB. Each point represents a single nucleotide polymorphism (SNP), plotted by chromosomal position (x-axis) and strength of association (–log10(P), y-axis). SNPs in blue indicate genome-wide significant associations. Gene annotations below each panel are based on the human genome reference (hg19), indicating the location of nearby genes within the region (17.8–18.8 Mb on chromosome 8).

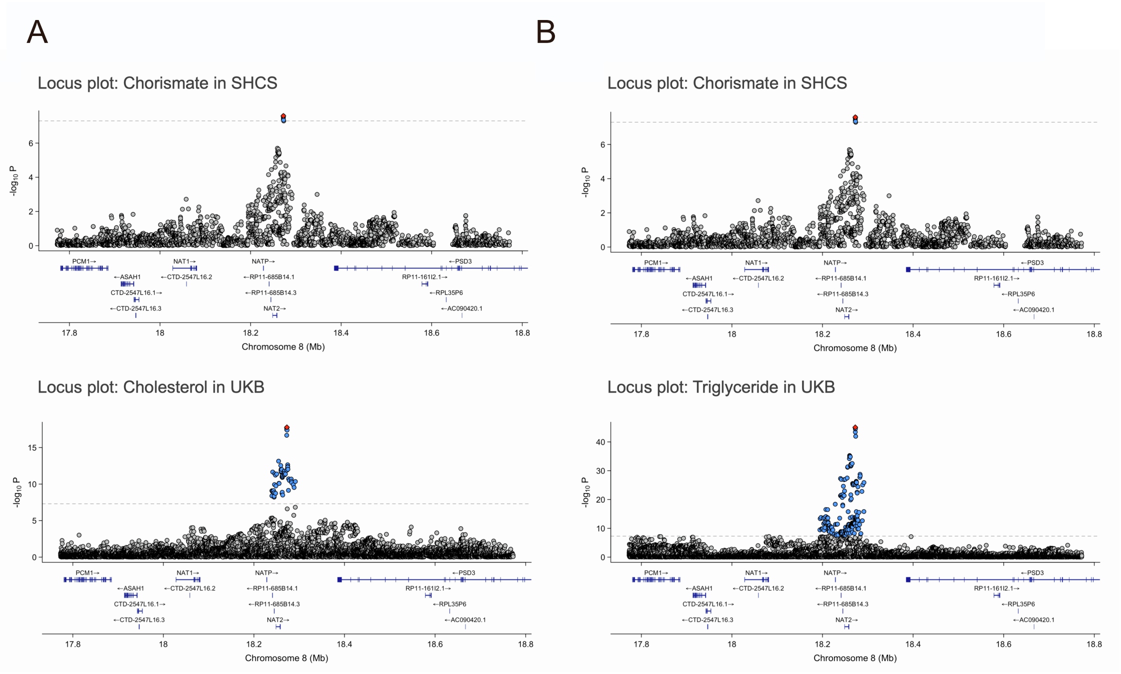

Figure S9. Causal effects of specific metabolites on cliical biomarkers of organ function using Mendelian randomization. (A) Forest plot of causal estimates for $C_{12}H_{22}O_{10}$ (matching HMDB entry for 2-O-L-fucopyranosyl-galactose) across a panel of clinical outcomes. (B) Forest plot of causal estimates for $C_{10}H_{10}O_6$ (matching HMDB entry for Chorismate). Error bars represent 95% confidence intervals. Effect sizes (α coefficients) are shown along with p-values for each outcome. Abbreviations: CHOL, cholesterol; GGT, gamma-glutamyl transferase; AST, aspartate aminotransferase; TRIG, triglycerides; ALB, albumin; CREAT, creatinine; HDL, high-density lipoprotein.

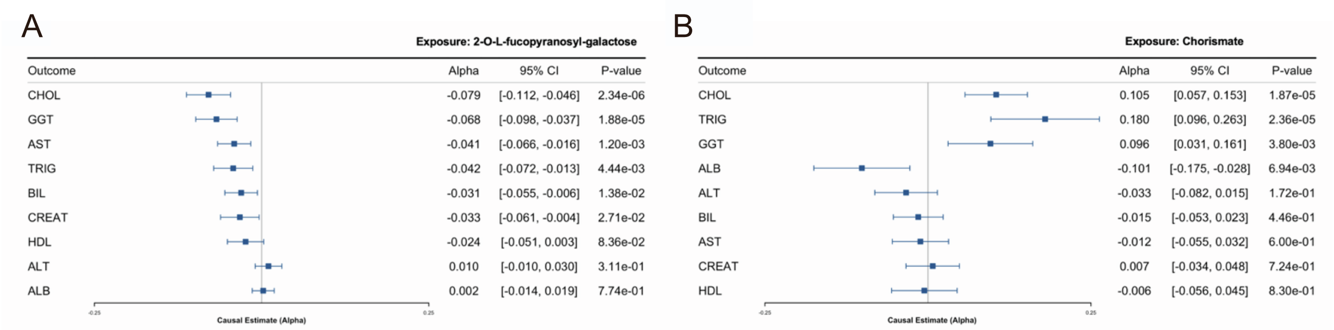

Supplementary methods

Aging-related comorbidities definition

Estimated glomerular filtration Rate (eGFR) and chronic kidney disease (CKD)
We defined eGFR and CKD in line with the approach reported by Roth et al. in the Swiss HIV Cohort Study [1]. Participants were classified as cases if they fulfilled the CKD definition at or prior to the time of metabolomic profiling.

Liver fibrosis
Liver fibrosis was evaluated using aspartate aminotransferase–to–platelet ratio index (APRI) [2]. Participants were classified as having liver fibrosis if they had an APRI score greater than 1.5 on three consecutive measurements at or prior to the time of metabolomic profiling.

Diabetes mellitus type II
Diabetes was defined as a fasting blood glucose concentration greater than 7 mmol/L or a blood glucose value exceeding 11.1 mmol/L on two consecutive measurements, irrespective of sampling conditions. Individuals receiving oral antidiabetic therapy or insulin were also classified as having diabetes. Participants were classified as cases if they fulfilled the criteria described above at or prior to the time of metabolomic profiling.

Cardiovascular disease (CVD)
CVD included myocardial infarction, stroke, and major invasive cardiovascular procedures, including carotid endarterectomy, coronary angioplasty or stenting, coronary artery bypass grafting, interventions on peripheral arteries, and heart transplantation. Participants were considered cases if at least one cardiovascular event occurred at or prior to the time of metabolomic profiling.

Key circulating biomarkers
We examined biomarkers reflecting liver, kidney, and cardiovascular function. Liver-related markers included alanine transaminase (ALT), aspartate transaminase (AST), albumin, bilirubin, and gamma-glutamyltransferase (GGT). Kidney function markers included serum creatinine and eGFR. Lipid parameters included high-density lipoproteins (HDL), low-density lipoprotein (LDL), and triglycerides. Given that most participants had several biomarker measurements from routine clinical visits, we calculated the mean of all values collected within the six months prior to metabolomic profiling.

Metabolite data generation
Metabolites were extracted from fasting plasma by methanol precipitation: samples were thawed on ice (30–60 min), then 20 μL plasma was mixed with 180 μL 80% methanol (room temperature), vortexed for 15 seconds, incubated for 1 h at 4 $^{\circ}$C, and

centrifuged at $> 14{,}000, g$ for 15 min (room temperature); 100 μL of the supernatant was transferred to a fresh tube and stored at $-20,^{\circ}$C until shipment. Metabolome extracts were analyzed using flow-injection time-of-flight mass spectrometry (Agilent 6550 QTOF, negative mode) [3]. This untargeted approach scans for metabolites between 50-1000 Dalton, detecting over 10,000 distinct mass-to-charge features. High mass accuracy ($\sim$ 1 mDalton) and isotopic pattern analysis enable the identification of several hundred metabolites across diverse chemical classes. While isomers with identical composition cannot be resolved due to the lack of chromatographic separation, the method can still determine elemental compositions and compound classes. For each formula, we enumerated the metabolites listed by the Human Metabolome Data base 4.0 [4]. Samples were analyzed sequentially (~1000/day) to minimize batch effects.

Description of the SHCS metabolome

The human metabolome database version 5.0 (HMDB) [5] was downloaded (on 06/12/2023) and parsed to categorize metabolites into specific superpathways (lipids/lipid-like molecules, amino acids/peptides, carbohydrates, and nucleosides/nucleotides). We assigned the metabolites not categorized in the previously described classes to the "xenobiotic" superpathway if they were part of any of the following categories from various databases provided by the MBROLE3 server [6]: KEGG DRUG, the Therapeutic Target (TTD), PharmGKB, MeSH (key: "pharmacological role"), PathBank (keys: "Drug Action","Drug Metabolism"), biological role CHEBI (keys: "xenobiotic", "drug", "pesticide", "flavor", "food", "additive","toxin", "inhibitor", "poison", "pharma", "vitamin", "nutrient","agent","polluant","environment","industry","chemical") databases. Any remaining metabolites that did not fit into the aforementioned categories were grouped under the term "others". Any remaining metabolites that did not fit into the aforementioned categories were grouped under the term "others".

Genome-wide association studies of metabolite levels

GWAS were performed using REGENIE v3.2.5.3 through a two steps procedure to account for population structure [7]. In summary, the initial step involves creating a whole-genome regression model to predict individual traits using genetic data. This is done by employing the leave-one-chromosome-out (LOCO) approach. We used a set of high-quality genotyped variants that passed all the following filters: MAF $\geq$ 0.05, genotyping rate = 100%, Hardy–Weinberg equilibrium test p-value $> 10^{-6}$, and linkage-disequilibrium (LD) pruning (50 variant windows, 5 sliding windows and $r^2 < 0.8$), resulting in 619,116 variants. In the second step, the phenotypic predictions obtained through LOCO were used as offsets for conducting variant association analysis through standard linear regression. For this part, we retained variants with a MAF $\geq$ 0.05 and missing rate < 0.1, leading to approximately 5.95 million SNPs. Following data processing and quality control, GWAS analyses using linear regression for metabolites were conducted, accounting for covariates including age at time of metabolomic

profiling, sex assigned at birth, smoking status, genotyping batches, and the first 10 genetic principal components.
To identify conditionally independent SNPs from the GWAS, GCTA-COJO [8][9] was used with the following parameters: maf 0.05, cojo-p 5 $x10^{-8}$, cojo-wind 5000, cojo-collinear 0.9.

Identification of potential novel associations and novel loci
To determine whether the associations had been previously reported, we queried significant genetic variants–metabolite associations ($P < 5 \times 10^{-8}$) or stronger study-specific p-value threshold) from recent GWAS. The inclusion criteria for these studies were: utilization of mass spectrometry platforms, focus on plasma or blood metabolites, cohorts of European ancestry, and publicly available data. Based on these criteria, we included seven relevant studies: Shin et al. (2014) [10], Long et al. (2017) [11], Lotta et al. (2021) [12], Hysi et al. (2022) [13], Yin et al. (2022) [14], Schlosser et al. (2023) [15], and Chen et al (2023) [16]. The cohort size, the number of metabolites tested and the genetic variants reported in these studies are summarized in the Additional files.
Unlabeled metabolites were discarded from the analysis. All the metabolite names from different sources were harmonized to HMDB identifiers. The matching was performed using the HMDB ID when available in the study. If the HMDB ID was not provided, we relied on InChIKey or the formatted compound name, incorporating all known synonyms from the database. To support reproducibility and facilitate future use, we've compiled the code used for mapping metabolites to HMDB IDs into an R package (https://github.com/mariamaitoumelloul/MetaboMapper). Metabolites that could not be matched to an HMDB ID were removed. To improve the matching analysis, we used Additional file 4 from Chen et al.(2023), which includes harmonized names for studies published prior to its release and mentioned above. Using our matching approach, we merged this table with our study and the more recent Schlosser et al.(2023) study, and completed the unmatched entries that could be matched through our analysis. Combined with our study, the seven metabolite QTLs (mQTLs) studies under investigation had 2935 unique harmonized metabolites measured. In our study, 314 metabolites were detected in common with these mQTL studies (see Additional file). The significant variant–metabolite pairs we identified were classified as "known association" if these genetic variants were the same or in strong linkage disequilibrium ($r^2 > 0.8$ using the 1kGP) with previously reported variants associated with the same metabolite. The remaining associations were considered as "potentially novel".

Extended eQTL Colocalization Procedures

To investigate whether genome-wide significant GWAS variants influenced gene expression, we performed expression quantitative trait locus (eQTL) colocalization analyses. Briefly, for each significant GWAS result, a genomic region was defined by extracting SNPs within a +-500 kbp window of the most significant hit. The local expression quantitative trait locus (cis-eQTL) association signals within this genomic

window were then extracted from the 2023 eQTL Catalogue [17]. This catalogue contains uniformly processed eQTL data from numerous, large studies, including GTEx [18] and TwinsUK [19]. Finally, the SNPs from the GWAS and the association hits from each eQTL for each gene were then colocalized using the coloc v.5.2.3 package [20]. This package applies Bayesian statistical tests to determine the probability that in this genomic window, there is 1) one genetic signal associated with the GWAS hit, 2) one genetic signal associated with the eQTL tested, and 3) that these two colocalize. The posterior probability for each GWAS x eQTL x gene combination was recorded. The metabolites that had PP.H4 ≥ 0.8 (posterior probabilities of two traits share one causal SNP) with eQTL were considered to pass the colocalization test.

References

[1] J. A. Roth, G. Radevski, C. Marzolini, A. Rauch, H. F. Günthard, R. D. Kouyos et al., "Cohort-derived machine learning models for individual prediction of chronic kidney disease in people living with human immunodeficiency virus: A prospective multicenter cohort study," J Infect Dis, vol. 224, no. 7, pp. 1198–1208, 2021, doi:10.1093/infdis/jiaa236.

[2] C. T. Wai, J. K. Greenson, R. J. Fontana, J. D. Kalbfleisch, J. A. Marrero, and H. S. Conjeevaram, "A simple noninvasive index can predict both significant fibrosis and cirrhosis in patients with chronic hepatitis C," Hepatology, vol. 38, no. 2, pp. 518–526, 2003, doi: 10.1053/jhep.2003.50346.

[3] T. Fuhrer and N. Zamboni, "High-throughput discovery metabolomics," *Curr Opin Biotechnol*, vol. 31, pp. 73–78, 2015, doi: 10.1016/j.copbio.2014.08.006

[4] D. S. Wishart *et al.*, "HMDB 4.0: The human metabolome database for 2018," *Nucleic Acids Research*, vol. 46, no. D1, pp. D608–D617, 2018, doi: 10.1093/nar/gkx1089.

[5] D. S. Wishart *et al.*, "HMDB 5.0: The human metabolome database for 2022," *Nucleic Acids Research*, vol. 50, pp. D622–D631, 2022-, doi: 10.1093/nar/gkab1062.

[6] J. Lopez-Ibañez, F. Pazos, and M. Chagoyen, "MBROLE3: Improved functional enrichment of chemical compounds for metabolomics data analysis," *Nucleic Acids Res*, vol. 51, pp. W305–W309, 2023, doi: 10.1093/nar/gkad405.

[7] J. Mbatchou *et al.*, "Computationally efficient whole-genome regression for quantitative and binary traits," *Nat Genet*, vol. 53, no. 7, pp. 1097–1103, 2021, doi: 10.1038/s41588-021-00870-7.

[8] J. Yang, S. H. Lee, M. E. Goddard, and P. M. Visscher, "GCTA: A tool for genome-wide complex trait analysis," *Am J Hum Genet*, vol. 88, no. 1, pp. 76–82, 2011, doi: 10.1016/j.ajhg.2010.11.011.

[9] J. Yang *et al.*, “Conditional and joint multiple-SNP analysis of GWAS summary statistics identifies additional variants influencing complex traits,” *Nat Genet*, vol. 44, no. 4, pp. 369–375, S1–3, 2012, doi: 10.1038/ng.2213.

[10] S.-Y. Shin *et al.*, “An atlas of genetic influences on human blood metabolites,” *Nat Genet*, vol. 46, no. 6, pp. 543–550, 2014, doi: 10.1038/ng.2982.

[11] T. Long *et al.*, “Whole-genome sequencing identifies common-to-rare variants associated with human blood metabolites,” *Nat Genet*, vol. 49, no. 4, pp. 568–578, 2017, doi: 10.1038/ng.3809.

[12] L. A. Lotta *et al.*, “A cross-platform approach identifies genetic regulators of human metabolism and health,” *Nat Genet*, vol. 53, no. 1, pp. 54–64, 2021, doi: 10.1038/s41588-020-00751-5.

[13] P. G. Hysi *et al.*, “Metabolome genome-wide association study identifies 74 novel genomic regions influencing plasma metabolites levels,” *Metabolites*, vol. 12, no. 1, p. 61, 2022, doi: 10.3390/metabo12010061.

[14] X. Yin *et al.*, “Integrating transcriptomics, metabolomics, and GWAS helps reveal molecular mechanisms for metabolite levels and disease risk,” *Am J Hum Genet*, vol. 109, no. 10, pp. 1727–1741, 2022, doi: 10.1016/j.ajhg.2022.08.007.

[15] P. Schlosser *et al.*, “Genetic studies of paired metabolomes reveal enzymatic and transport processes at the interface of plasma and urine,” *Nat Genet*, vol. 55, no. 6, pp. 995–1008, 2023, doi: 10.1038/s41588-023-01409-8.

[16] Y. Chen *et al.*, “Genomic atlas of the plasma metabolome prioritizes metabolites implicated in human diseases,” *Nat Genet*, vol. 55, no. 1, pp. 44–53, 2023, doi: 10.1038/s41588-022-01270-1.

[17] N. Kerimov *et al.*, “eQTL catalogue 2023: New datasets, x chromosome QTLs, and improved detection and visualisation of transcript-level QTLs,” *PLOS Genetics*, vol. 19, no. 9, p. e1010932, 2023, doi: 10.1371/journal.pgen.1010932.

[18] F. Aguet *et al.*, “Genetic effects on gene expression across human tissues,” *Nature*, vol. 550, no. 7675, pp. 204–213, 2017, doi: 10.1038/nature24277.

[19] S. Verdi *et al.*, “TwinsUK: The UK adult twin registry update,” *Twin Res Hum Genet*, vol. 22, no. 6, pp. 523–529, 2019, doi: 10.1017/thg.2019.65.

[20] C. Wallace and C. Giambartolomei, “Coloc: Colocalisation tests of two genetic traits.” 2012. doi: 10.32614/CRAN.package.coloc.
